# A recurrent variant in *LIM2* causes an isolated congenital sutural/lamellar cataract in a Japanese family

**DOI:** 10.1080/13816810.2022.2090010

**Published:** 2022-06-23

**Authors:** Vanita Berry, Kaoru Fujinami, Kiyofumi Mochizuki, Takeshi Iwata, Nikolas Pontikos, Roy A. Quinlan, Michel Michaelides

**Affiliations:** a Department of Genetics, UCL Institute of Ophthalmology, University College London, London, UK; b Moorfields Eye Hospital NHS Foundation Trust, London, UK; c Laboratory of Visual Physiology, Division of Vision Research, National Institute of Sensory Organs, National Hospital Organization, Tokyo Medical Centre, Tokyo, Japan; d Department of Ophthalmology, Gifu University Graduate School of Medicine, Gifu, Japan; e Division of Molecular and Cellular Biology, National Institute of Sensory Organs, National Hospital Organization Tokyo Medical Center, Tokyo, Japan; f Department of Biosciences, University of Durham, Durham, UK

**Keywords:** Autosomal dominant congenital sutural/lamellar cataract, WES, *LIM2*, *COL11A1*

## Abstract

**Background:**

Genetically determined cataract is both clinically and molecularly highly heterogeneous. Here, we have identified a heterozygous variant in the lens integral membrane protein LIM2, the second most abundant protein in the lens, responsible for congenital sutural/lamellar cataract in a three-generation Japanese family.

**Methods:**

Whole exome sequencing (WES) was undertaken in one affected and one unaffected individual from a family with autosomal dominant congenital cataract to establish the underlying genetic basis.

**Results:**

A recurrent missense variant LIM2: c.388C>T; p.R130C was identified and found to co-segregate with disease. In addition, one variant COL11A1:c.3788C>T of unknown significance (VUS) was also identified.

**Conclusions:**

We report a variant in LIM2 causing an isolated autosomal-dominant congenital sutural/lamellar cataract in a Japanese family. This is the first report of a LIM2 variant in the Japanese population. Hence, we expand the mutation spectrum of LIM2 variants in different ethnic groups.

## Introduction

Pediatric cataract is a genotypically and phenotypically heterogeneous condition causing visual impairment either from birth or in early infancy. The full spectrum of congenital cataract-causing genes can be found here https://cat-map.wustl.edu/ (1,2). In this study, we have identified two heterozygous variants, *LIM2* p.R130C and *COL11A1* p.P1263 L, in a Japanese family with non-syndromic congenital cataract. As previously reported, *LIM2* c.388C>T; p.R130C is predicted to be pathogenic and damaging at both a bioinformatic and a structural level (3), while *COL11A1* p.P1263 L is of unknown significance (VUS). It is a rare variant in many ethnicities, but in the Japanese population, *COL11A1* p.P1263 L has an allele frequency of 0.006. *LIM2* variants have been associated with autosomal dominant, autosomal recessive, and age-related cataracts. Collagen’s importance in ocular health is well-established, with disease-causing variants in collagen encoding genes being associated with high myopia, glaucoma, congenital cataracts, and syndromes such as Stickler syndrome and Marshall syndrome. Pathogenic variants in *COL11A1* are known to cause both the Marshall and Stickler type 2 syndromes, rare autosomal dominant disorders (4) along with other ocular, orofacial, auditory, and skeletal manifestations (5). Common variants in *COL11A1* are associated with angle-closure glaucoma in Asian populations (6). Here, for the first time, we report a heterozygous variant in *LIM2* gene causing a non-syndromic autosomal-dominant congenital sutural/lamellar cataract in the Japanese population.

## Material and methods

### Phenotyping

**Figure 1.**
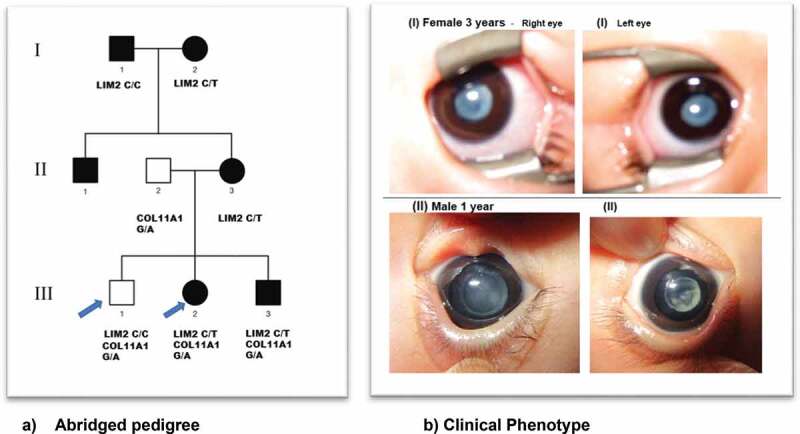
(a) Abridged pedigree with lamellar/sutural congenital cataract. Squares and circles symbolize males and females, respectively. Open and filled symbols indicate unaffected and affected individuals, respectively. The arrows indicate the family members who participated in the WES analysis and were sequenced to show segregation; (b) (I) III:2 depicts bilateral lamellar cataract and in (II) III-3 with sutural cataract in left eye and lamellar cataract in the right eye.

The Japanese family studied was identified through the proband attending the Department of Ophthalmology, Gifu University, and gDNA sample extraction was performed in the National Institute of Sensory Organs, National Hospital Organization, Tokyo Medical Centre, Tokyo, Japan. Local ethics committee approval [R18-029] was obtained and all individuals taking part gave written informed consent and underwent a full ophthalmic examination. All affected individuals were diagnosed as having isolated bilateral congenital cataract with either lamellar or sutural/lamellar phenotype (Figure 1).

#### Whole exome sequencing (WES) and bioinformatics analysis

Genomic DNA was extracted from EDTA sequestered blood samples using the Nucleon II DNA Extraction Kit (Scotlab Bioscience, Strathclyde, Scotland, UK). The DNA samples were sequenced at Macrogen Europe. Exon capture and target enrichment was performed using the SureSelectXT Human All Exon V6 post (Agilent, Santa Rosa, CA, USA). Paired-end sequencing was performed on an Illumina HiSeq 2500 high-throughput sequencer, generating mean exome coverage of 50×. Raw data in fastq format were analyzed using the Phenopolis bioinformatics platform (7), and data were aligned to the GRCh37/hg19 human reference sequence using Burrows–Wheeler Aligner (BWA-MEM) and then marked duplicates with GATK*’s Mark Duplicates. Variants and indels were called according to GATK (version 3.5.0) best practices (joint variant calling followed by variant quality score recalibration). The moderately or highly damaging variants were then annotated using the Variant Effect Predictor (VEP) (8). Variants with a sequencing depth of less than 20X were filtered out. Variants were then filtered to only contain novel variants which were absent in public control databases Kaviar (https://db.systemsbiology.net/kaviar/) (9) and Genome Aggregation Database (gnmAD, (https://gnomad.broadinstitute.org/) or rare variants (GnomAD allele frequency <0.0001). Recurrent mutations were identified from 357 known cataract genes (https://cat-map.wustl.edu/) and predicted to be moderately or highly damaging (CADD >15). The filtered variants were then ordered on CADD score with the highest at the top. Further bioinformatic validations were done on the varsome platform (varsome.com).

### Sanger sequencing

Direct Sanger sequencing was performed to validate the variant identified by whole exome sequencing. Genomic DNA was amplified by PCR using GoTaq 2X master mix (AB gene; Thermo Scientific, Epsom, UK) and *COL11A1* specific primers Forward primer: *cagcatcaagcctgcatatt*; Reverse primer: *aaggcagcaggacttctttg* and *LIM2* - Forward primer: *tcaacccctatcctcactcct*; Reverse primer: *gtgggacaccctgtcatctt* were designed with http://bioinfo.ut.ee/primer3-10.4.0/. PCR conditions were as follows: 94– for 5°Cmin of initial denaturation followed by 30 cycles of amplification of 30 s at 94  denaturing, 30 s at 60°C annealing, and 45 s at 72°C for extending. After cleaning, the PCR products were reacted with BigDye Terminator v3.1, they were run on ABI 3730 Genetic Analyzer (both from Applied Biosystems, Foster City, CA, USA) and analyzed using SeqMan Pro (version 8.0.2 from DNASTAR) sequence analysis. After validating the variant, segregation was performed on all the available family members.

## Results

A three-generation Japanese pedigree with 6 affected, 1 unaffected, and 2 spouses presented with bilateral lamellar/sutural congenital cataract (Figure 1). Individual III-2, a 3-year-old female had bilateral lamellar cataract. Individual III-3, a 2-month old male was diagnosed with sutural cataract in the left eye and lamellar opacities in the right eye. Their mother II-3, grandmother I-2 and grandfather I-1, aged 30, 55, and 56 years, respectively, all had bilateral cataract.

One unaffected individual (III-1) and one affected (III-2) were sequenced by WES and analyzed using the Phenopolis genetic variant analysis pipeline. All of the variants were filtered by allele frequency databases included in Phenopolis: Gnomad genomes, Kaviar, the Japanese IRD database, the HGD, and the Tommo database. In III-1, from a total of 2349 rare coding variants, 27 variants from known genes remained, with the top scoring variant for CADD (score of 32) a VUS heterozygous variant NM_001854.4 c.3788C>T; p.P1263 L in exon of 50/67 of *COL11A1*. In III-2, 27/2420 variants remained, with the top scoring variant for CADD (score of 35.1) a rare heterozygous variant NM_001161748.2 c.388C>T; p.R130C in exon 4 of *LIM2* and also the second variant found was *COL11A1*:c.3788C>T; p.P1263 L.

**Figure 2.**
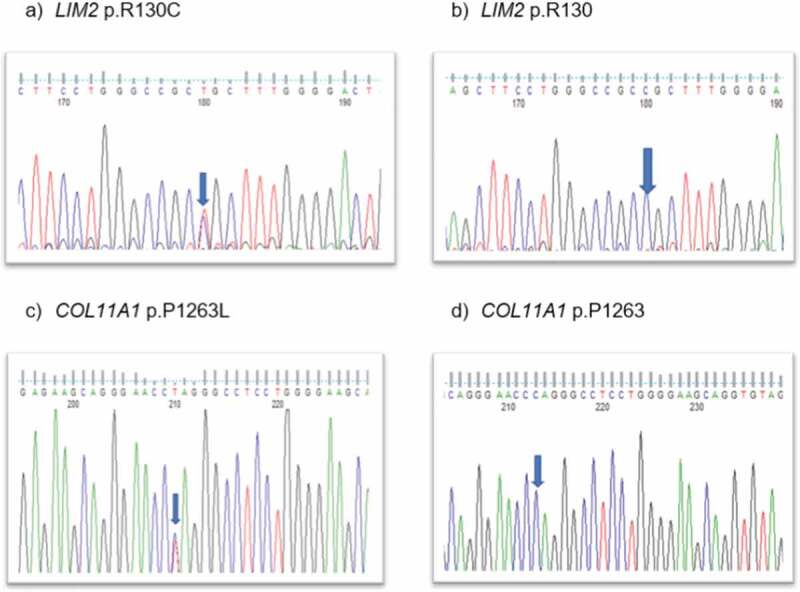
Sequence analysis—(a) *LIM2*–missense variant c.388c>t in affected member, (b) *LIM2* wild type, (c) *COL11A1*-missense variant at c.3788c>t in affected and unaffected family members, and (d) wild-type *COL11A1.*

The *LIM2* variant was predicted to be damaging (Table 1). Direct Sanger sequencing confirmed these variants (Figure 2), with the *LIM2* substitution co-segregating in I-2, II-3, III-2, and III-3. The disease-causing LIM2 (c.388C>T) variant is passed down by I-2 in the family, though I-1 is also affected (for unknown reasons) despite having the normal LIM2 allele. The *COL11A1* variant:c.3788C>T; p.P1263 L was found to be in II-2, III-1, III-2, and III-3. This is a rare variant in all populations with a highest allele frequency of 0.0000276 (https://gnomad.broadinstitute.org/), except in the Japanese population with a highest frequency of 0.006 (https://togovar.biosciencedbc.jp/doc/datasets/gem_j_wgahttps://togovar.biosciencedbc.jp/doc/datasets/gem_j_wga).

**Table 1.** Pathogenicity scores of variants in *COL11A1* and *LIM2* genes causing sutural/lamellar cataract.

Genes	Genomic pos./Exon	HGVSp	MutationTaster/verdict	PROVEAN	REVEL	GERP	CADD	SIFT	DANN
*LIM2*	Chr19q13.4/Ex-4/5	p.R130C	Disease-causing/0.81-Pathogenic	Damaging /0.74	Pathogenic/0.96	4.73	35.1	Damaging	0.999
*COL11A1*	Chr1p21.1/Ex-50/67	p.P1263L	Uncertain Significance/0.81	Damaging /0.9	Pathogenic/0.89	5.4	32.0	Damaging	0.997

Protein Variation Effect Analyzer (PROVEAN) is to predict the functional consequences of amino acid substitutions and indels; Rare Exome Variant Ensemble Learner (REVEL) tool is to predict the pathogenicity of missense variants based on a combination of scores from 13 individual tools; Genomic Evolutionary Rate Profiling (GERP) NR corresponds to the neutral rate conservation score of the site; Combined Annotation Dependent Depletion (CADD) is a score for the deleteriousness of a variant. A CADD score >15 is considered damaging; Sorting Intolerant From Tolerant (SIFT) score (0.0–0.05) to check the deleteriousness of the amino acid substitution on the protein function; * indicates the truncated protein.

## Discussion

Here, we report heterozygous variants in *LIM2* and *COL11A1* causing non-syndromic autosomal dominant congenital sutural/lamellar cataract in a three-generation Japanese pedigree. Pathogenicity scores of variants in *LIM2* and *COL11A1* are shown in Table 1.

Both LIM2 and COL11A1 are expressed in ocular tissues. LIM2 is a 173-amino-acid membrane protein, also known as MP19, with four transmembrane domains, two extracellular loops, a cytoplasmic loop, and cytoplasmic amino and carboxyl termini (17). LIM2 is the second most abundant integral membrane protein present in the ocular lens fiber cells of vertebrates (18). It has both an adhesive role (19,20) and supports the formation and organization of the lens fiber junctions (21). To date (including the present study), 12 variants have been identified to cause autosomal-dominant, recessive, and age-related cataracts. Half of the variants have been at p.R130C, across various ethnic groups, thereby representing a hotspot in the protein (Table 2). The R130C substitution is located in the second extracellular loop of the LIM2 protein and is likely to perturb membrane trafficking and fiber cell–cell communication (3). The first extracellular loop contains a tetraspanin topology motif that contains cysteine (WGLWCC), and disulphide bridges are important to the function of this loop and therefore the proximity of another cysteine in the extracellular loop 2 (R130C) could interfere with folding of the tetraspanin homology domain.

**Table 2.** *LIM2* Variants causing cataractogenesis to date.

No.	Exon	HGVSc	HGVSp	Inheritance	Origin	Phenotype	Ocular defects	Reference
1.	Ex2/5	c.57G>A	p.L19L	Complex	China	Age-related cortical		(10)
2.	Ex2/5	c.67A>C	p.M23L	Complex	China	Age-related cortical		(10)
3.	Ex3/5	c.233 G>A	p.G78D	AR	Pakistan	Nuclear		(11)
4.	Ex3/5	c.313T>G	p.F105V	AR	Iraq	Presenile, cortical, sutural		(12)
5.	Ex4/5	c.385C>T	p.R129C	AD	Spain	-		(13)
6.	Ex4/5	c.388C>T	p.R130C	AD	UK/Czechia	Nuclear pulverulent		(3)
7.	Ex4/5	c.388C>T	p.R130C	AD	China	Membranous	Nystagmus, amblyopia	(14)
8.	Ex4/5	c.388C>T	p.R130C	AD	China	Variable (nuclear, lamellar, pulverulent)	Elongated Axial Length, Myopia	(15)
9.	Ex4/5	c.388C>T	p.R130C	AD	Spain	Nuclear		(13)
10.	Ex4/5	c.388C>T	p.R130C	AD	Spain	Lamellar		(13)
11.	Ex4/5	**c.388C>T**	**p.R130C**	**AD**	**Japan**	**sutural/Lamellar**		**Present Study**
12.	Ex4/5	c.462G>A	p.G154E	AR	India	Congenital	Nystagmus, amblyopia	(16)

Furthermore, Collagen type XI, alpha 1 (*COL11A1*) another disease-causing variant is found in this family. Collagens are fibrous structural proteins involved in the construction of skin, cartilage, bone, eye, and other tissues. Collagen type XI, alpha 1, minor fibrillar collagen, is a member of this large diverse group consisting of 20 genes to date (22,23). Recently, COL11A1 has been recruited as novel biomarker and a key player in cancer (24). COL11A1 is expressed in the mouse lens including the fiber cells (25) and in the zebrafish model, COL11A1, its knockdown affected lens and optic cup diameter during early development (26) indicative of a functional role in the lens.

Pathogenic variants in *COL11A1* have been linked to specific genetic disorders of the connective tissue, namely Marshall syndrome (27) and Stickler syndrome type 2 (28,29) and contribute to bilateral ophthalmological abnormalities, as well as systemic effects, such as a distinctive facial appearance, hearing loss, and joint problems (30) Typical ophthalmological findings include congenital high myopia, abnormal vitreous, glaucoma, retinal detachment, and cataracts. Recently, a pathogenic variant in *COL11A1* in a large family has been linked to non-syndromic hearing loss (31).

Here, we report variants in *COL11A1* and *LIM2* in congenital cataract. Although no direct evidence for LIM2 and COL11A1 interaction has been documented in the literature, both genes are expressed during the early development of the eye indicative of the congenital cataract phenotypes identified in the family here. A specific interaction between COL11A1 and LIM2 has yet to be reported. Collagens are located extracellularly between differentiating fiber cells during lens development, so it is reasonable to expect a congenital cataract phenotype including for COL11A1 (32–36). Intriguingly with collagen mutations, it can be observed that unilateral lens cataract develops (33) in the affected individual and can even be absent between generations (36) demonstrating that phenotypic variability is a known complication for congenital cataract (37). Why the highly damaging *COL11A1* mutation in the Japanese resulted in an unaffected phenotype remains unknown; however, further study is needed to explore the pathogenic consequences of both of these variants in the lens to understand the underlying molecular and functional mechanisms, but this also applies to other collagen mutations associated with congenital cataract.

## References

[1] Shiels A, Hejtmancik JF. Biology of inherited cataracts and opportunities for treatment. Annu Rev Vis Sci. 2019;5:123–49. doi:10.1146/annurev-vision-091517-034346. 31525139 PMC6791712

[2] Berry V, Georgiou M, Fujinami K, Quinlan R, Moore A, Michaelides M. Inherited cataracts: molecular genetics, clinical features, disease mechanisms and novel therapeutic approaches. Br J Ophthalmol. 2020;104(10):1331–37. doi:10.1136/bjophthalmol-2019-315282. 32217542

[3] Berry V, Pontikos N, Dudakova L, Moore AT, Quinlan R, Liskova P, Michaelides M. A novel missense mutation in LIM2 causing isolated autosomal dominant congenital cataract. Ophthalmic Genet. 2020;41(2):131–34. doi:10.1080/13816810.2020.1737950. 32202185

[4] Richards AJ, McNinch A, Martin H, Oakhill K, Rai H, Waller S, Treacy B, Whittaker J, Meredith S, Poulson A, et al. Stickler syndrome and the vitreous phenotype: mutations in COL2A1 and COL11A1. Hum Mutat. 2010;31(6):E1461–71. doi:10.1002/humu.21257. 20513134

[5] Snead MP, Yates JR. Clinical and Molecular genetics of Stickler syndrome. J Med Genet. 1999;36(5):353–59. 10353778 PMC1734362

[6] Vithana EN, Khor C-C, Qiao C, Nongpiur ME, George R, Chen L-J, Do T, Abu-Amero K, Huang CK, Low S, et al. Genome-Wide association analyses identify three new susceptibility loci for primary angle closure glaucoma. Nat Genet. 2012;44(10):1142–46. doi:10.1038/ng.2390. 22922875 PMC4333205

[7] Pontikos N, Yu J, Moghul I, Withington L, Blanco-Kelly F, Vulliamy T, Wong TLE, Murphy C, Cipriani V, Fiorentino A, et al. Phenopolis: an open platform for harmonization and analysis of genetic and phenotypic data. Bioinformatics. 2017;33(15):2421–23. doi:10.1093/bioinformatics/btx147. 28334266

[8] McLaren W, Gil L, Hunt SE, Riat HS, Ritchie GRS, Thormann A, Flicek P, Cunningham F, et al. The Ensembl variant effect predictor. Genome Biol. 2016;17:122. 27268795 10.1186/s13059-016-0974-4 PMC4893825

[9] Glusman G, Caballero J, Mauldin DE, Hood L, Roach JC. Kaviar: accessible system for testing SNV novelty. Bioinformatics. 2011;27:3216–17. doi:10.1093/bioinformatics/btr540. 21965822 PMC3208392

[10] Zhou Z, Wang B, Hu S, Zhang C, Ma X, Qi Y. Genetic variations in GJA3, GJA8, LIM2, and age-related cataract in the Chinese population: a mutation screening study. Mol Vis. 2011;17:621–26. 21386927 PMC3049737

[11] Irum B, Khan SY, Ali M, Kaul H, Kabir F, Rauf B, Fatima F, Nadeem R, Khan AO, Al Obaisi S, et al. Mutation in LIM2 is Responsible for Autosomal Recessive Congenital Cataracts. PLoS One. 2016;11(11):e0162620. doi:10.1371/journal.pone.0162620. 27814360 PMC5096708

[12] Pras E, Levy-Nissenbaum E, Bakhan T, Lahat H, Assia E, Geffen-Carmi N, Frydman M, Goldman B, Pras E. A missense mutation in the LIM2 gene is associated with autosomal recessive presenile cataract in an inbred Iraqi Jewish family. Am J Hum Genet. 2002;70(5):1363–67. doi:10.1086/340318. 11917274 PMC447612

[13] Fernández-Alcalde C, Nieves-Moreno M, Noval S, Peralta JM, Montaño VEF, Del Pozo Á, Santos-Simarro F, Vallespín E. Molecular and genetic mechanism of non-syndromic congenital cataracts. Mutat Screening Span Families Genes. 2021;12(4). doi:10.3390/genes12040580. PMC8072554 33923544

[14] Pei R, Liang P-F, Ye W, Li J, Ma J-Y, Zhou J. A novel mutation of LIM2 causes autosomal dominant membranous cataract in a Chinese family. Int J Ophthalmol. 2020;13(10):1512–20. doi:10.18240/ijo.2020.10.02. 33078099 PMC7511386

[15] Wang X, Qin Y, Abudoukeremuahong A, Dongye M, Zhang X, Wang D, Li J, Lin Z, Yang Y, Ding L, et al. Elongated axial length and myopia-related fundus changes associated with the Arg130Cys mutation in the LIM2 gene in four Chinese families with congenital cataracts. Ann Transl Med. 2021;9(3):235. doi:10.21037/atm-20-4275. 33708862 PMC7940952

[16] Ponnam SPG, Ramesha K, Tejwani S, Matalia J, Kannabiran C. A missense mutation in LIM2 causes autosomal recessive congenital cataract. Mol Vis. 2008;14:1204–08. 18596884 PMC2442473

[17] Arneson ML, Louis CF. Structural arrangement of lens fiber cell plasma membrane protein MP20. Exp Eye Res. 1998;66(4):495–509. doi:10.1006/exer.1998.0477. 9593642

[18] Mulders JW, Voorter CE, Lamers C, de Haard-Hoekman WA, Montecucco C, van de Ven WJ, Bloemendal H, de Jong WW. MP17, a fiber-specific intrinsic membrane protein from mammalian eye lens. Curr Eye Res. 1988;7(2):207–19. doi:10.3109/02713688808995750. 3371069

[19] Shiels A, King JM, Mackay DS, Bassnett S. Refractive defects and cataracts in mice lacking lens intrinsic membrane protein-2. Invest Ophthalmol Vis Sci. 2007;48(2):500–08. doi:10.1167/iovs.06-0947. 17251442

[20] Fotiadis D, Hasler L, Müller DJ, Stahlberg H, Kistler J, Engel A. Surface tongue-and-groove contours on lens MIP facilitate cell-to-cell adherence. J Mol Biol. 2000;300(4):779–89. doi:10.1006/jmbi.2000.3920. 10891268

[21] Tenbroek E, Arneson M, Jarvis L, Louis C. The distribution of the fiber cell intrinsic membrane proteins MP20 and connexin46 in the bovine lens. J Cell Sci. 1992;103(Pt 1):245–57. doi:10.1242/jcs.103.1.245. 1331134

[22] Ricard-Blum S. The collagen family. Cold Spring Harb Perspect Biol. 2011;3(1):a004978. doi:10.1101/cshperspect.a004978. 21421911 PMC3003457

[23] Spranger J. The type XI collagenopathies. Pediatr Radiol. 1998;28(10):745–50. doi:10.1007/s002470050459. 9799295

[24] Nallanthighal S, Heiserman JP, Cheon D-J. Collagen Type XI Alpha 1 (COL11A1): a Novel Biomarker and a Key Player in Cancer. Cancers. 2021;13(5). doi:10.3390/cancers13050935. PMC7956367 33668097

[25] Lin X, Li H, Yang T, Liu X, Fan F, Zhou X, Luo Y. Transcriptomics Analysis of Lens from Patients with Posterior Subcapsular Congenital Cataract. Genes. 2021;12(12). doi:10.3390/genes12121904. PMC8702110 34946854

[26] Yonkers M, Oxford J. Knockdown of Collagen 11A1 decreases zebrafish lens and optic cup diameter during early development. Invest Ophthalmol Vis Sci. 2013;54(15):465.

[27] Annunen S, Körkkö J, Czarny M, Warman ML, Brunner HG, Kääriäinen H, Mulliken JB, Tranebjaerg L, Brooks DG, Cox GF, et al. Splicing mutations of 54-bp exons in the COL11A1 gene cause Marshall syndrome, but other mutations cause overlapping Marshall/Stickler phenotypes. Am J Hum Genet. 1999;65(4):974–83. doi:10.1086/302585. 10486316 PMC1288268

[28] Majava M, Hoornaert KP, Bartholdi D, Bouma MC, Bouman K, Carrera M, Devriendt K, Hurst J, Kitsos G, Niedrist D, et al. A report on 10 new patients with heterozygous mutations in the COL11A1 gene and a review of genotype-phenotype correlations in type XI collagenopathies. Am J Med Genet A. 2007;143A(3):258–64. doi:10.1002/ajmg.a.31586. 17236192

[29] Rose PS, Levy HP, Liberfarb RM, Davis J, Szymko-Bennett Y, Rubin BI, Tsilou E, Griffith AJ, Francomano CA. Stickler syndrome: clinical characteristics and diagnostic criteria. Am J Med Genet A. 2005;138A(3):199–207. doi:10.1002/ajmg.a.30955. 16152640

[30] Stickler GB, Belau PG, Farrell FJ, Jones JD, Pugh DG, Steinberg AG, Ward LE. Hereditary progressive arthro-ophthalmopathy. Mayo Clin Proc. 1965;40:433–55. 14299791

[31] Booth KT, Askew JW, Talebizadeh Z, Huygen PLM, Eudy J, Kenyon J, Hoover D, Hildebrand MS, Smith KR, Bahlo M, et al. Splice-Altering variant in COL11A1 as a cause of nonsyndromic hearing loss DFNA37. Genet Med. 2019;21(4):948–54. doi:10.1038/s41436-018-0285-0. 30245514 PMC6431578

[32] Reis LM, Semina EV. Genetic landscape of isolated pediatric cataracts: extreme heterogeneity and variable inheritance patterns within genes. Hum Genet. 2019;138(8–9):847–63. doi:10.1007/s00439-018-1932-x. 30187164 PMC6401332

[33] Kjellström U, Martell S, Brobeck C, Andréasson S. Autosomal recessive Stickler syndrome associated with homozygous mutations in the COL9A2 gene. Ophthalmic Genet. 2021;42(2):161–69. doi:10.1080/13816810.2020.1861309. 33356723

[34] Nau S, McCourt EA, Maloney JA, Van Hove JL, Saenz M, Jung JL. COL4A1 mutations in two infants with congenital cataracts and porencephaly: an ophthalmologic perspective. J Aapos. 2019;23(4):246–48. doi:10.1016/j.jaapos.2019.04.003. 31128271

[35] Mark PR, Torres-Martinez W, Lachman RS, Weaver DD. Association of a p.Pro786leu variant in COL2A1 with mild spondyloepiphyseal dysplasia congenita in a three-generation family. Am J Med Genet A. 2011;155A(1):174–79. doi:10.1002/ajmg.a.33762. 21204228

[36] Griffith AJ, Gebarski SS, Shepard NT, Kileny PR. Audiovestibular phenotype associated with a COL11A1 mutation in Marshall syndrome. Arch Otolaryngol Head Neck Surg. 2000;126(7):891–94. doi:10.1001/archotol.126.7.891. 10889003

[37] Shiels A, Hejtmancik JF. Inherited cataracts: Genetic mechanisms and pathways new and old. Exp Eye Res. 2021;209:108662. doi:10.1016/j.exer.2021.108662. 34126080 PMC8595562

